# Network Analysis of Metabolome and Transcriptome Revealed Regulation of Different Nitrogen Concentrations on Hybrid Poplar Cambium Development

**DOI:** 10.3390/ijms25021017

**Published:** 2024-01-13

**Authors:** Shuang Zhang, Lina Cao, Ruhui Chang, Heng Zhang, Jiajie Yu, Chunming Li, Guanjun Liu, Junxin Yan, Zhiru Xu

**Affiliations:** 1 College of Life Science, Northeast Forestry University, Harbin 150040, China; shuangzhang_1994@126.com (S.Z.); crh1107@126.com (R.C.); 2 State Key Laboratory of Tree Genetics and Breeding, Northeast Forestry University, Harbin 150040, China; caolina0901@126.com (L.C.); zhaghengphd@163.com (H.Z.); 13514554189@163.com (J.Y.); lichunming@nefu.edu.cn (C.L.); liuguanjun2013@nefu.edu.cn (G.L.); 3 College of Landscape Architecture, Northeast Forestry University, Harbin 150040, China

**Keywords:** nitrogen, cambium, metabolite, transcript, hybrid poplar

## Abstract

Secondary development is a key biological characteristic of woody plants and the basis of wood formation. Exogenous nitrogen can affect the secondary growth of poplar, and some regulatory mechanisms have been found in the secondary xylem. However, the effect of nitrogen on cambium has not been reported. Herein, we investigated the effects of different nitrogen concentrations on cambium development using combined transcriptome and metabolome analysis. The results show that, compared with 1 mM NH_4_NO_3_ (M), the layers of hybrid poplar cambium cells decreased under the 0.15 mM NH_4_NO_3_ (L) and 0.3 mM NH_4_NO_3_ (LM) treatments. However, there was no difference in the layers of hybrid poplar cambium cells under the 3 mM NH_4_NO_3_ (HM) and 5 mM NH_4_NO_3_ (H) treatments. Totals of 2365, 824, 649 and 398 DEGs were identified in the M versus (vs.) L, M vs. LM, M vs. HM and M vs. H groups, respectively. Expression profile analysis of the DEGs showed that exogenous nitrogen affected the gene expression involved in plant hormone signal transduction, phenylpropanoid biosynthesis, the starch and sucrose metabolism pathway and the ubiquitin-mediated proteolysis pathway. In M vs. L, M vs. LM, M vs. HM and M vs. H, differential metabolites were enriched in flavonoids, lignans, coumarins and saccharides. The combined analysis of the transcriptome and metabolome showed that some genes and metabolites in plant hormone signal transduction, phenylpropanoid biosynthesis and starch and sucrose metabolism pathways may be involved in nitrogen regulation in cambium development, whose functions need to be verified. In this study, from the point of view that nitrogen influences cambium development to regulate wood formation, the network analysis of the transcriptome and metabolomics of cambium under different nitrogen supply levels was studied for the first time, revealing the potential regulatory and metabolic mechanisms involved in this process and providing new insights into the effects of nitrogen on wood development.

## 1. Introduction

Poplar, a perennial wild woody plant, has emerged as a vital component of the terrestrial ecosystem in the northern hemisphere, distinguished by its distinctive genome, physiology and developmental characteristics [1]. Poplar is widely used in the pulp and paper industry, afforestation land and bio-energy materials, which hold significant ecological implications and economic value. As a model plant, it has been instrumental in unraveling the molecular mechanisms governing secondary meristem development [2]. The vascular cambium, a meristem cell population supporting the secondary growth of plant stems, originates from procambium, which, in turn, derives from the stem apex meristem (SAM). Daughter cells of the cambium are recruited into the secondary phloem or secondary xylem, where they differentiate into functional cells. Over time, this process of secondary growth leads to radial thickening of the stems and a larger organizational structure [3]. The transition from primary growth to secondary growth occurs approaching the stem apex, with the initiation of secondary growth observed in the initial three internodes. Once the vascular cambium is established, it can maintain activity for decades or even centuries [2].

The formation of phloem outward and xylem inward is generated by the division and differentiation of the vascular cambium, with its activity influencing the development of both xylem and phloem. In poplar, the overexpression of *REVOLUTA* and mutated forms of *PtrHB7* (uninhibited by miRNA165/166) involved in initiating vascular cambium lead to the emergence of ectopic vascular cambium and the subsequent development of ectopic xylem [4,5]. The overexpression of *ARK1* in poplar results in the inhibition of cambium activity, delayed xylem development and the loss of phloem fiber cells. However, overexpressing *ARK2* promotes vascular cambium formation while inhibiting the differentiation of secondary xylem [6,7]. With the inhibited expression of *PttWOX4a/b*, a reduction in the activity of vascular cambium division and the production of secondary xylem are observed [8]. These findings indicate that vascular cambium activity is essential for wood formation.

The regulation of vascular cambium development involves various factors, including hormones [9], signaling peptides and receptor kinases [10], along with post-translational regulation [11] and epigenetic modification [12]. Auxin (Aux) not only plays a central role in the regulation of procambium initiation and maintenance but also has been demonstrated to be crucial in the vascular cambium of *Arabidopsis*. It has been shown that a local high Aux signal can define stem cell organizers in vascular cambium, and an elevated exogenous Aux signal can promote cell differentiation into a xylem vessel element and induce the production of marker genes in phloem cells and cambial cells, respectively [13]. In addition to Aux, the development of vascular cambium is influenced by cytokinin (CK), gibberellin (GA) and brassinolide (BR), each of which exhibits distinct effects [9,14,15]. *PtrCLE20* is transcribed in the developing xylem, but its peptide is mainly distributed in the vascular cambium. In poplar, the overexpression of *PtrCLE20* results in a significant reduction in cambium and secondary xylem, suggesting that the signaling peptides of PtrCLE20 could inhibit the activity of vascular cambium and thus regulate the formation of xylem [16]. Ubiquitin receptor protein DA1 regulates the stability of the WOX4 protein, which is cambium’s key transcription factor, after ubiquitination modification and then regulates xylem differentiation by changing vascular cambium activity in poplar [11]. In the apical meristem, *Arabidopsis REPRESSOR OF WUSCHEL1* (*ROW1*) regulates the histone methylation modification of the *WOX5* promoter (H3K4me3) to inhibit *WOX5* transcription and further regulates the development of the apical meristem [17].

Fundamentally, cambium acts like the SAM as a source of meristem cells to sustain the indefinite growth of cells. In the central region of the SAM, the *WUSCHEL* (*WUS*) gene is required to specify the fate of meristem cells [6]. In *Arabidopsis*, a feedback loop is formed by the major genes *CLAVATA3* (*CLV3*) and *WUS* to maintain the stability of stem cells in the root meristem. Additionally, the inhibition of stem cell differentiation is enacted by *SHOOTMERISTEMLESS* (*STM*) and related homeobox genes [18]. Nevertheless, it is noteworthy that mutants lacking CLV3 activity exhibit the presence of stem cells, indicating that CLV3 is not imperative for the identification of stem cells [18]. A microarray analysis of gene expression during secondary growth found that key or homologous genes regulating the SAM are also expressed in the cambial region. For example, homeodomain-leucine zipper and *KANADI* transcription factors responsible for adaxial–abaxial patterning in the SAM are also expressed in the cambium region of *Populus* [19]. Schrader et al. identified different subdomains in the 60 μm cambium region of poplar, including phloem mother cells, cambium stem cells and xylem mother cells, based on the coordinated pattern of gene expression [19]. In hybrid aspen, it was identified that many genes are differentially regulated during the transition to secondary growth, among which the expression levels involved in secondary wall formation and lignin biosynthesis change significantly. In addition, there are some interesting transcription factors and other potential signal transduction genes that might participate in the transition from primary growth to secondary growth [20,21]. For example, *PtaRHE1* (zinc finger protein) has a pronounced expression in cambium and has a potential role in vascular development [21]. In addition, the gene expression levels of acid invertase, glutamine synthetase, cytochrome P450, cinnamyl alcohol dehydrogenase, 4-coumarate: CoA ligase and glucosyltransferase significantly change during the progressive transition from primary to secondary growth [21].

Nitrogen (N) is a fundamental requirement for the normal growth of plants, influencing the majority of physiological developmental processes [22]. The impact of N on plants is observable across all facets, ranging from the cellular level to the entire plant [23]. The assimilation of N into amino acids significantly influences carbon (C) and N metabolism [24]. Genes involved in C metabolism play a coordinated role in regulating N availability [25]. The secondary growth of trees accounts for the main part of the stem biomass, and the differentiation of cambium involves the formation of lignified secondary cell walls [26,27]. The main structural components of the secondary cell wall are cellulose, lignin and hemicellulose, which are the final forms of carbon [26,28]. A wood structure is established during xylem generation as a consequence of cambium activity [29]. In hybrid poplars (*P. trichocarpa* Torr. and Gray. × *deltoides* Bartr. ex Marsh), elevated nitrogen levels were found to cause a 17% widening and an 18% shortening of fiber cells [24]. At present, it has been demonstrated that specific genes exhibit a differential response to high N availability in stems. For example, the expression level of *CCoAOMT* (caffeoyl-CoA O-methyltransferase) is regulated by N, as well as other proteins that may be related to cell wall development, such as leucine-rich repeating proteins [30]. In tobacco (*Nicotiana tabacum* L.), the interplay between phenylalanine metabolism and nitrate availability was elucidated through a nitrate-reductase-deficient mutant [Nia30(145)], providing both metabolic and transcriptional evidence of lignin formation [31].

Several studies have collaboratively unveiled the influence of N on the composition and structure of the secondary cell wall in trees, where the secondary cell wall is a result of cambium activity. Although existing research predominantly delves into the impact of nitrogen on the structure and composition of secondary xylem, there is a notable gap in understanding the effects of nitrogen on secondary growth from the standpoint of cambium development. *P.* × *xiaohei* T. S. Hwang et Liang, a hybrid poplar obtained by crossing *P. simonii* and *P. nigra*, exhibits desirable traits such as rapid growth, drought resistance and resilience to pests and diseases. It serves as an excellent fast-growing green tree species in the sandy and arid regions of northern China and provides an ideal material for investigating the mechanisms of wood formation [32]. In this study, five different concentrations of N were supplied to *P.* × *xiaohei* T. S. Hwang et Liang. A comparative analysis of the effects of various N concentrations on the wood structure was conducted, and the potential mechanisms were explored through cambium transcriptome and metabolome analysis. This study represents the inaugural attempt to analyze the impact of N on poplar’s secondary growth from the cambium perspective. It not only offers novel insights into the effects of different N concentrations on wood development but also reveals potential regulatory networks involved in this process.

## 2. Results

### 2.1. Effects of Different Nitrogen Concentrations on Growth and Wood Property of Poplar

**Figure 1:**
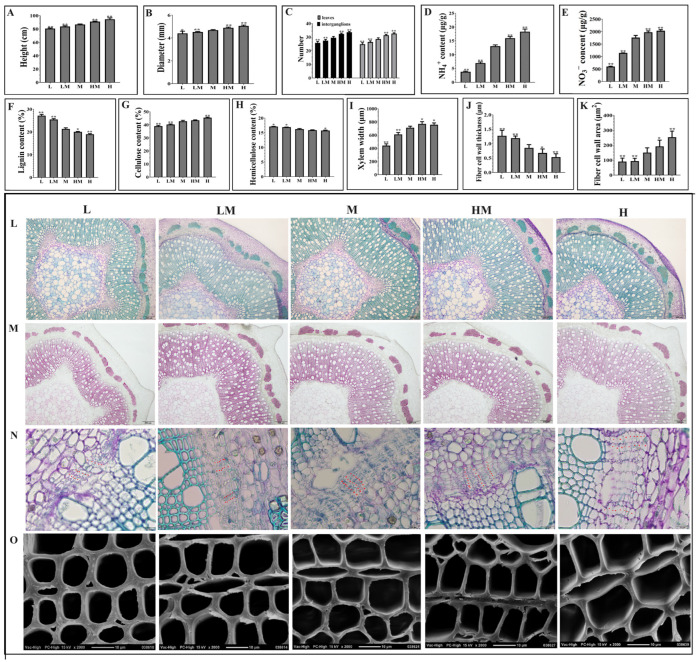
Effects of nitrogen on poplar growth and wood development. (**A**) Plant height. (**B**) Ground diameter. (**C**) Number of internodes and leaves. (**D**) NH_4_^+^ content. (**E**) NO_3_^−^ content. (**F**) Lignin content. (**G**) Cellulose content. (**H**) Hemicellulose content. (**I**) Xylem width. (**J**) Fiber cell wall thickness. (**K**) Fiber cell wall area. (**L**) Toluidine blue staining, bar: 200 μm. (**M**) Phloroglucinol–HCl staining, bar: 200 μm. (**N**) Layers of cambial cells, bar: 20 μm. (**O**) SEM, bar: 10 μm. The notations L, LM, M, HM and H represent 0.15 mM NH_4_NO_3_, 0.3 mM NH_4_NO_3_, 1 mM NH_4_NO_3_, 3 mM NH_4_NO_3_ and 5 mM NH_4_NO_3_, respectively. * and ** indicate 0.01 < *p* < 0.05 and *p* < 0.01, respectively.

Under different N concentrations, the plant height, ground diameter and number of internodes and leaves of hybrid poplar increased with the increase in the N concentration (Figure 1A–C). At the same time, the content of NH_4_^+^ and NO_3_^−^ was higher in HM and H conditions than in L and LM conditions (Figure 1D,E). It was found that the cellulose content was decreased under the conditions of L and LM, with increased lignin and hemicellulose content, compared with that in the M treatment. Conversely, under the HM and H treatments, those contents exhibited the opposite trend (Figure 1F–H). Microscopic observation (Figure 1L–O) showed that the L and LM treatments resulted in a decreased xylem width, deepened phloroglucinol-HCl staining (indicating increased lignin content), a declined number of cambium cell layers and thickened fiber cell walls compared to the M treatment. Conversely, the H and HM treatments exhibited the opposite effects (except for the number of cambial cell layers, which showed no difference compared to the M treatment). Figure 1I–K present a quantitative analysis of Figure 1L,O. The findings indicate that, under the L and LM treatments, the wood width and fiber cell area were significantly lower than those under the M treatment, and the fiber cell wall thickness was significantly higher than that under the M treatment, whereas the H and HM treatments exhibited the opposite effects. The data of these indexes are listed in Table S1.

### 2.2. Transcriptome Sequencing and Annotation

Under the L, LM, M, HM and H treatments, 15 cDNA libraries were constructed for the cambium of poplar. As shown in Table S2, among all samples, transcriptome sequencing generated 53,454,620–72,450,762 raw reads, and Q30 ranged from 93.09% to 94.05%. The proportion of clean reads in raw reads ranged from 91.20% to 94.76%, and the clean reads were mapped with poplar reference genomes. The results show that 42,944,848–57,819,332 reads were successfully mapped with the reference genomes, accounting for 86.33% to 87.91% of the clean reads. Therefore, the transcriptome sequencing data had high quality. The clean reads were annotated using the KEGG, Nr, Swissport, TrEMBL, KOG, GO and Pfam databases, and the annotation results are presented in Table 1. The database annotation and expression pattern results mentioned above lay the foundation for a more in-depth analysis of transcriptome changes in the cambium under the five treatments. The correlation analysis for the samples showed that the samples in the interclass were highly correlated (Figure S1A), indicating that the samples had good repeatability. Principal component analysis showed that there were differences among the five treatments, and samples in the interclass could be clustered together (Figure S1B), indicating that different treatments had an impact on cambial transcription levels and that interclass samples had high repeatability.

### 2.3. Analysis of DEGs

The number of differentially expressed genes (DEGs) was obtained under different N concentrations. In the groups M versus (vs.) L, M vs. LM, M vs. HM and M vs. H, 2365, 824, 649 and 398 DEGs were screened, respectively. In the comparisons M vs. L, M vs. LM, M vs. HM and M vs. H, there were 1183, 615, 236 and 89 up-regulated DEGs and 1182, 209, 413 and 309 down-regulated DEGs, respectively (Figure S2A–D). The cluster analysis of DEG expression patterns showed that DEG expression patterns were different among groups, and intraclass samples could be clustered together (Figure S2E), indicating that different N affected the expression patterns of cambium genes.

**Figure 2:**
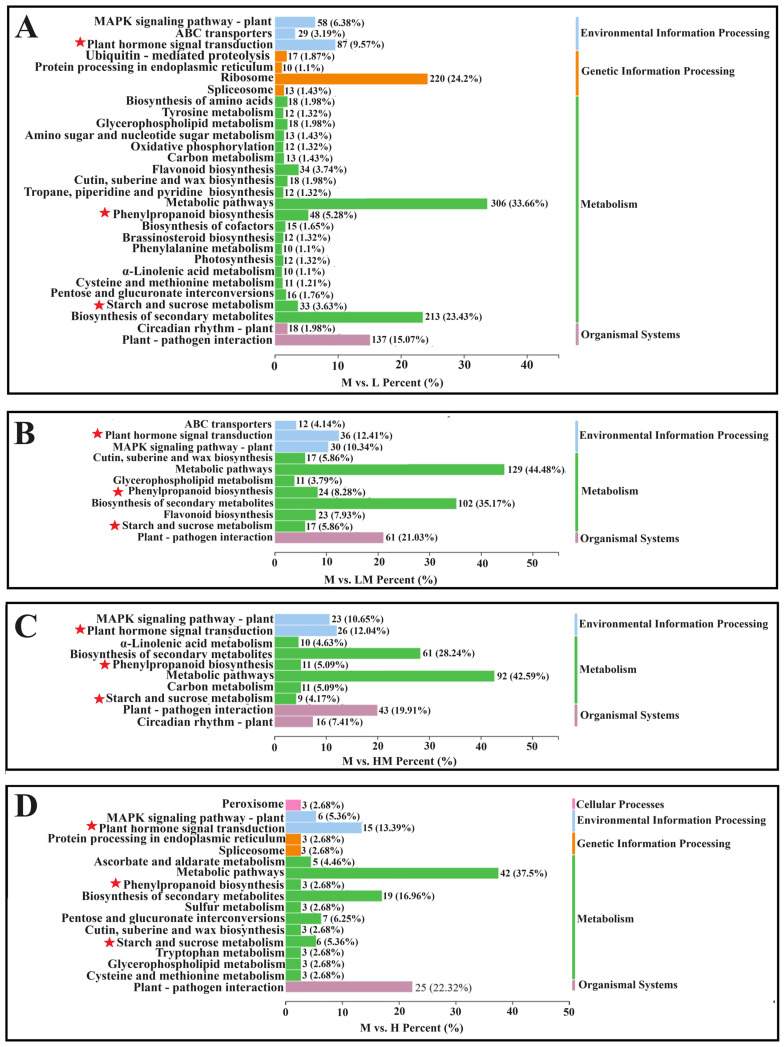
Enrichment analysis of DEGs in KEGG databases. (**A**) M vs. L, (**B**) M vs. LM, (**C**) M vs. HM and (**D**) M vs. H DEGs KEGG enrichment column. The pathways of attention are indicated by red stars. The notations M vs. L, M vs. LM, M vs. HM and M vs. H represent 1 mM NH_4_NO_3_ versus 0.15 mM NH_4_NO_3_, 1 mM NH_4_NO_3_ versus 0.3 mM NH_4_NO_3_, 1 mM NH_4_NO_3_ versus 3 mM NH_4_NO_3_ and 1 mM NH_4_NO_3_ versus 5 mM NH_4_NO_3_, respectively.

To elucidate the function of DEGs in each comparison, GO and KEGG pathway enrichment analyses were performed. As shown in Figure S3A–D, the top five significantly enriched biological process subcategories were GO:0002181 (cytoplasmic translation), GO:0000028 (ribosomal small subunit assembly), GO:0042274 (ribosomal small subunit biogenesis), GO:0009698 (phenylpropanoid metabolic process) and GO:0051703 (biological process involved in intraspecies interaction between organisms) in M vs. L; GO:0010345 (suberin biosynthetic process), GO:0009698 (phenylpropanoid metabolic process), GO:0009699 (phenylpropanoid biosynthetic process), GO:0000160 (phosphorelay signal transduction system) and GO:0044550 (secondary metabolite biosynthetic process) in M vs. LM; GO:0071456 (cellular response to hypoxia), GO:0036294 (cellular response to decreased oxygen levels), GO:0071453 (cellular response to oxygen levels), GO:0001666 (response to hypoxia) and GO:0036293 (response to decreased oxygen levels) in M vs. HM; and GO:0009611 (response to wounding), GO:0031099 (regeneration), GO:0071456 (cellular response to hypoxia), GO:0036294 (cellular response to decreased oxygen levels) and GO:0071453 (cellular response to oxygen levels) in M vs. H. For the cell component subcategory, GO:0022626 (cytosolic ribosome), GO:0044391 (ribosomal subunit), GO:0022625 (cytosolic large ribosomal subunit), GO:0015934 (large ribosomal subunit) and GO:0022627 (cytosolic small ribosomal subunit) in M vs. L; GO:0031225 (anchored component of membrane) and GO:0005615 (extracellular space) in M vs. LM; and GO:0016602 (CCAAT-binding factor complex) in M vs. H were significantly enriched. For the molecular function subcategory, GO:0019843 (rRNA binding), GO:0015293 (symporter activity), GO:0016682 (oxidoreductase activity, acting on diphenols and related substances as donors, oxygen as acceptor), GO:0016679 (oxidoreductase activity, acting on diphenols and related substances as donors) and GO:0070181 (small ribosomal subunit rRNA binding) in M vs. L; GO:0015293 (symporter activity), GO:0102756 (very-long-chain 3-ketoacyl-CoA synthase activity), GO:0016762 (xyloglucan: xyloglucosyl transferase activity), GO:0016682 (oxidoreductase activity, acting on diphenols and related substances as donors, oxygen as acceptor) and GO:0045486 (naringenin 3-dioxygenase activity) in M vs. LM; GO:0015141 (succinate transmembrane transporter activity), GO:0004097 (catechol oxidase activity), GO:0017077 (oxidative phosphorylation uncoupler activity), GO:0015131 (oxaloacetate transmembrane transporter activity) and GO:0015140 (malate transmembrane transporter activity) in M vs. HM; and GO:0016762 (xyloglucan: xyloglucosyl transferase activity), GO:0008447 (L-ascorbate oxidase activity), GO:0005249 (voltage-gated potassium channel activity), GO:0005267 (potassium channel activity) and GO:0022843 (voltage-gated monoatomic cation channel activity) in M vs. H were significantly enriched. The KEGG pathway enrichment analysis (Figure 2A–D) showed that, in pathways such as plant hormone signal transduction, phenylpropanoid biosynthesis, starch and sucrose metabolism, the biosynthesis of secondary metabolites and so on, they were enriched by the DEGs of the M vs. L, M vs. LM, M vs. HM and M vs. H groups. The above results indicate that the impact of exogenous N on cambium development was multifaceted.

**Figure 3:**
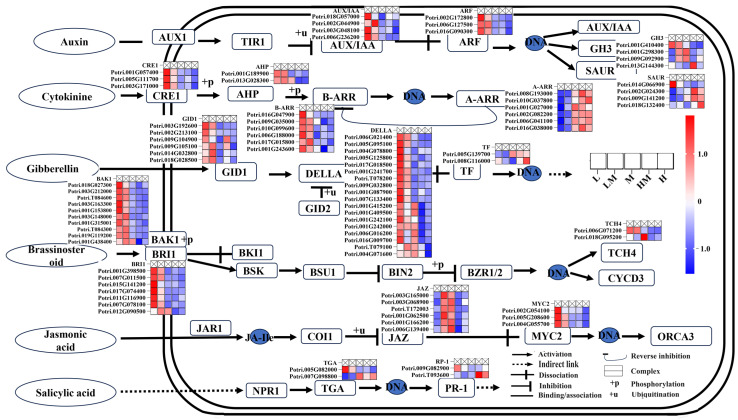
Response of genes to nitrogen in plant hormone signal transduction pathway. The gene expression patterns in the pathway are represented by the normalized (Z-score) FPKM and are embedded around the gene names, with scale bars listed on the right. AUX1: Auxin influx carrier; TIR1: Transport inhibitor response 1; AUX/IAA: Auxin-responsive protein IAA; ARF: Auxin response factor; GH3: Gretchen hagen3; SAUR: Small auxin up-regulated genes; CRE1: Cytokinin receptor; AHP: Histidine phosphotransfer proteins; B-ARR: Two-component response regulator ARR-B family; A-ARR: Two-component response regulator ARR-A family; GID1: GA insensitive dwarf 1; DELLA: group plant-specific transcriptional regulators of the GRAS family; GID2: GA-insensitive dwarf 2; TF: Phytochrome-interacting factor; BAK1: Brassinosteroid-insensitive 1-associated receptor kinase 1; BRI1: Brassinosteroid insensitive 1; BKI1: BRI1 kinase inhibitor 1; BSK: BR-signaling kinase; BSU1: BRI1 suppressor 1; BIN2: Brassinosteroid-insensitive 2; BZR1/2: Brassinosteroid r-sistant 1/2; TCH4: Xyloglucosyl transferase; CYCD3: Cyclin D3; JAR1: Jasmonic acid-amino synthetase; COI1: Coronatine-insensitive 1; JZA: Jasmonate ZIM domain-containing protein; MYC2: Transcription factor MYC2; ORCA2/3: AP2-domain DNA-binding protein; NPR1: Nonexpressor of pathogenesis-related genes 1; TGA: transcription factor TGA; PR1: Pathogenesis-related protein 1. The notations L, LM, M, HM and H represent 0.15 mM NH_4_NO_3_, 0.3 mM NH_4_NO_3_, 1 mM NH_4_NO_3_, 3 mM NH_4_NO_3_ and 5 mM NH_4_NO_3_, respectively.

To explain whether the regulation of hormones on cambium was affected by exogenous N application, the expression profile of DEGs in the plant hormone signal transduction pathway was analyzed. As shown in Figure 3, compared with the M treatment, the *AUX/IAA* and *ARF* family genes during Aux signaling responded to the L and LM treatments, and the changes in *GH3s* and *SAURs* were complex. In CK signal transduction, low N promotes CK signal transmission. For instance, a decrease in the expression level of *A-ARRs* was observed under L and LM conditions, diminishing its inhibitory effect on *B-ARRs* and thereby promoting the heightened expression of *B-ARRs* under L and LM. Additionally, the expression of *CRF1s* and *AHPs* was increased under L and LM, further facilitating the expression of *B-ARRs*. The components essential for GA signal transduction, including *GID1s*, *DELLAs* and *TFs*, were influenced to varying degrees by exogenous N. Specifically, *GID1s* respond solely to low N, whereas members of the *DELLA* family are regulated by both low and high N. In the BR signaling pathway, only the components *BAK1s* and *BRI1s*, necessary for the initial step, exhibit responsiveness to low N. The expression level of *JAZs* in the jasmonic acid signaling pathway decreased in the L, HM and H treatments, whereas the expression level of *MYC2s* increased only under the low N treatment. The changes in other genes were complex, suggesting that the genes involved in plant hormone signal transduction were affected to varying degrees by exogenous N.

**Figure 4:**
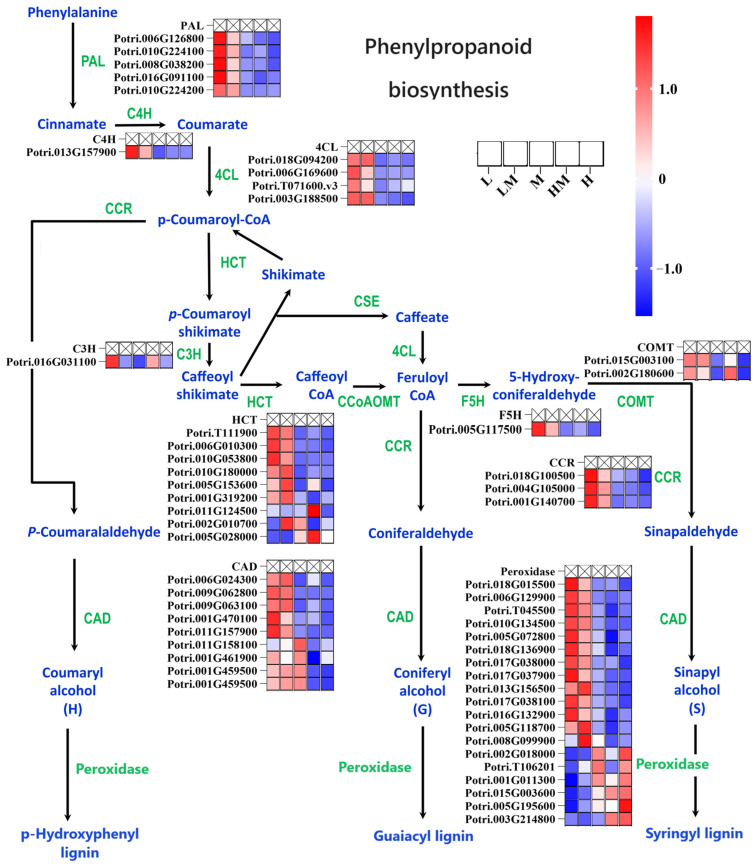
Gene responses to nitrogen in the phenylpropanoid biosynthesis pathway. The gene expression patterns in the pathway are represented by the normalized (Z-score) FPKM and are embedded around the gene names, with scale bars listed on the right. The path drawing was adapted from the KEGG website and previous publications [33]. PAL: Phenylalanine ammonialyase; C4H: Cinnamate 4-Hydroxylase; 4CL: 4-coumarate-CoA ligase; CCR: Cinnamoyl-CoA reductase; HCT: Shikimate O-hydroxy cinnamoyl transferase; C3H: Coumarate 3-hydroxylase; CSE: Caffeoyl shikimate esterase; CCoAOMT: Caffeoy-l CoA3-O-methyltransferase; F5H: Ferulate5-hydroxylase; COMT: Caffeic acid-3-O-methyltransferase; CAD: Cinnamoyl alcohol dehydrogenase. The notations L, LM, M, HM and H represent 0.15 mM NH_4_NO_3_, 0.3 mM NH_4_NO_3_, 1 mM NH_4_NO_3_, 3 mM NH_4_NO_3_ and 5 mM NH_4_NO_3_, respectively.

In order to clarify whether the effect of exogenous N on wood development began during cambium development, the gene expression profiles of the phenylpropanoid biosynthesis pathway were analyzed. As shown in Figure 4, compared with the M treatment, the *PAL*, *C4H*, *4CL*, *C3H*, *COMT*, *HCT*, *F5H*, *CCR*, *CAD* and *Peroxidase* family genes responded to exogenous N changes during the biosynthesis of phenylpropanoid. Compared to M, the expression levels of the *PAL*, *C4H*, *4CL*, *C3H*, *COMT*, *F5H* and *CCR* family genes were significantly increased under the L and LM treatments. Furthermore, members of the *CAD*, *HCT* and *Peroxidase* families showed different responses to exogenous N, but most of them were highly expressed under the L and LM treatments compared to those under M. These results indicate that the application of exogenous N affects the phenylpropanoid biosynthesis process in cambium.

**Figure 5:**
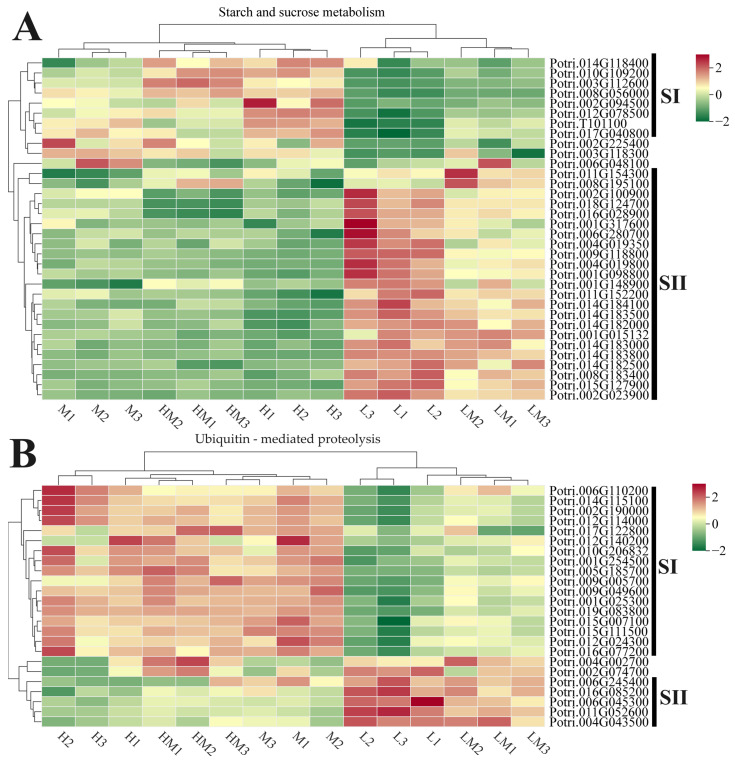
Gene responses to nitrogen in the starch and sucrose metabolism (**A**) and ubiquitin-mediated proteolysis (**B**) pathways. The gene expression patterns in the pathway are represented by the normalized (Z-score) FPKM, with scale bars and gene ID listed on the right. SI and SII represent the grouping of gene expression patterns. L1-3, LM1-3, M1-3, HM1-3 and H1-3, respectively, denote three biological replicates for the treatments with 0.15 mM NH_4_NO_3_, 0.3 mM NH_4_NO_3_, 1 mM NH_4_NO_3_, 3 mM NH_4_NO_3_ and 5 mM NH_4_NO_3_.

To explain whether the regulation of C content and ubiquitin degradation on cambium was affected by exogenous N application, the expression profiles of DEGs in the starch and sucrose metabolism and ubiquitin-mediated proteolysis pathways were analyzed. As shown in Figure 5A, the DEGs enriched in the starch and sucrose metabolism pathways exhibited two distinct expression patterns. Compared with the M treatment, the DEGs in the SI group demonstrated an elevated expression level with increasing N concentrations. In contrast, the DEGs in the SII group exhibited heightened expression only under the L and LM treatments, displaying no difference under HM and H conditions. Despite the non-enrichment of DEGs in the ubiquitin-mediated proteolysis pathway, we conducted an expression profile analysis (Figure 5B) of all genes involved in this pathway due to their regulatory role in phloem development. Only some genes responded to nitrogen, and their expression was specifically influenced by the L and LM treatments. Furthermore, these genes can be categorized into two groups: those with reduced expression under low nitrogen conditions (SI) and those induced under low nitrogen conditions (SII).

### 2.4. Metabolome Analysis

**Figure 6:**
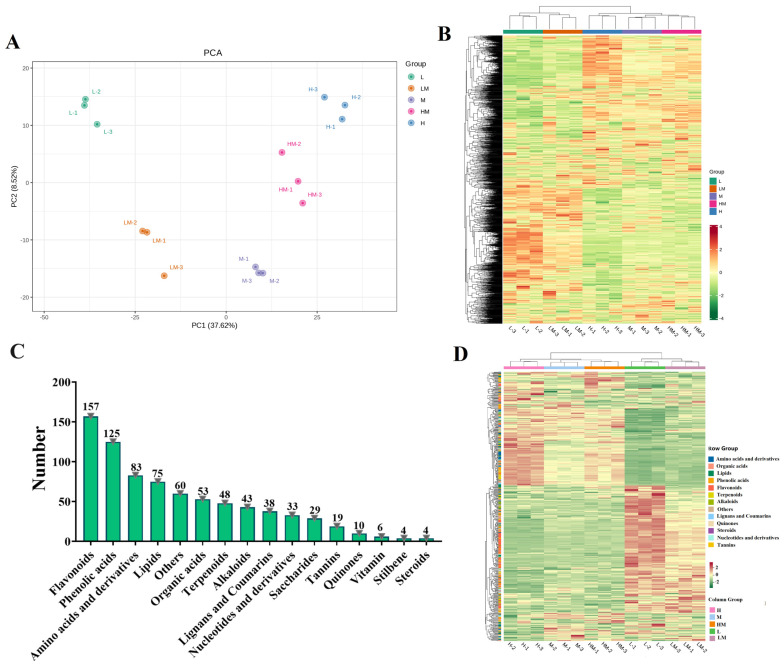
Analysis of the metabolites detected by the metabolome. (**A**) Principal component analysis of the samples. (**B**) Global content profiles of the metabolites of poplar cambium under different nitrogen concentration treatments. (**C**) Statistics of the metabolites in different subcategories. (**D**) Content changes in the DRMs of cambium under different nitrogen concentration treatments. L1-3, LM1-3, M1-3, HM1-3 and H1-3, respectively, denote three biological replicates for the treatments with 0.15 mM NH_4_NO_3_, 0.3 mM NH_4_NO_3_, 1 mM NH_4_NO_3_, 3 mM NH_4_NO_3_ and 5 mM NH_4_NO_3_.

A total of 1838 annotated metabolites were obtained via a metabolome detection system. A score scatter plot from the principal component analysis showed the isolation trend of five groups of samples according to the relative contents of the metabolites (Figure 6A). The overall content expression profile of metabolites showed significant changes in cambium under different treatments (Figure 6B). According to the threshold, differential metabolites (DRMs) were screened from 1838 metabolites, wherein 359, 190, 81 and 138 DRMs were screened in M vs. L (195 up-regulated, 164 down-regulated), M vs. LM (120 up-regulated, 70 down-regulated), M vs. HM (40 up-regulated, 70 down-regulated) and M vs. H (67 up-regulated and 71 down-regulated) (Table 2). DRMs were classified into 16 metabolic pathways, including flavonoids, phenolic acids, amino acids and derivatives, lipids, others, organic acids, terpenoids, alkaloids, lignans and coumarins, nucleotides and derivatives, saccharides, tannins, quinones, vitamin, stilbene, and steroids, with members of 157, 125, 83, 75, 60, 53, 48, 43, 38, 33, 29, 19, 10, 6, 4 and 4, respectively (Figure 6C). The changes in the DRM content showed that they were significantly influenced by different N concentrations (Figure 6D). Additionally, the expression profile of the DRMs, such as flavonoids, lignans and coumarins, and saccharides, indicated that substantial accumulation of most flavonoids and saccharides occurred under L and LM conditions compared to that under M (Figure S4A,B). The content of lignan and coumarin metabolites varied with the concentration of N (Figure S4C).

**Figure 7:**
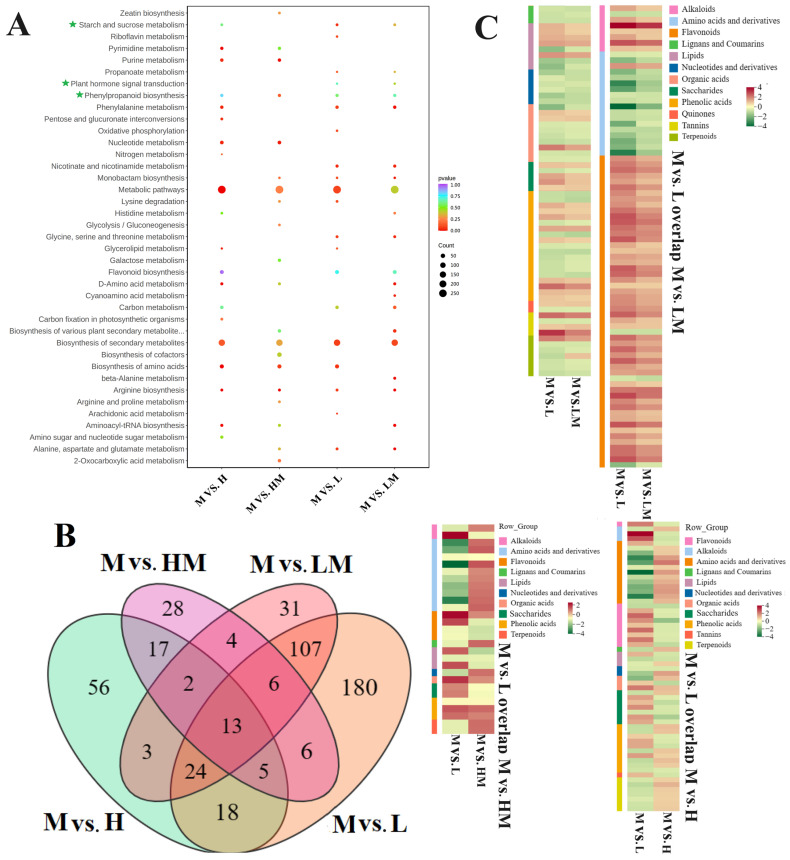
Differential metabolite analysis. (**A**) KEGG pathway enrichment of DRMs. (**B**) Statistics of overlapping metabolites among the four comparison groups. (**C**) DRMs of overlapping content analysis. Flavonoids are represented in pink, and phenolic acids are in blue. The scale bar is shown on the right side. The pathways of attention are indicated by green stars. The notations M vs. L, M vs. LM, M vs. HM and M vs. H represent 1 mM NH_4_NO_3_ versus 0.15 mM NH_4_NO_3_, 1 mM NH_4_NO_3_ versus 0.3 mM NH_4_NO_3_, 1 mM NH_4_NO_3_ versus 3 mM NH_4_NO_3_ and 1 mM NH_4_NO_3_ versus 5 mM NH_4_NO_3_, respectively.

The DRMs were further mapped to the KEGG pathways. The results reveal that the DRMs were enriched in plant hormone signal transduction, phenylpropanoid biosynthesis, starch and sucrose metabolism, the biosynthesis of secondary metabolites and so on (Figure 7A). The overlap analysis of the DRMs from the four comparison groups showed that there were 150 common DRMs in M vs. L and M vs. LM, 30 common DRMs in M vs. L and M vs. HM and 60 common DRMs in M vs. L and M vs. H (Figure 7B). The content analysis of DRMs mentioned above showed that the DRMs of flavonoids and saccharides were accumulated in the L and LM treatment conditions compared with the M treatment conditions, and the contents of DRMs in lignan and coumarin metabolites decreased. Compared with the M treatment conditions, the contents of DRMs in most saccharides (overlapping in M vs. L and M vs. H or overlapping in M vs. L and M vs. HM) increased in the L treatment conditions but decreased in the H treatment conditions, and the contents of most lignan and coumarin metabolite DRMs were reversed (Figure 7C).

### 2.5. Conjoint Analysis of Transcriptome and Metabolome

**Figure 8:**
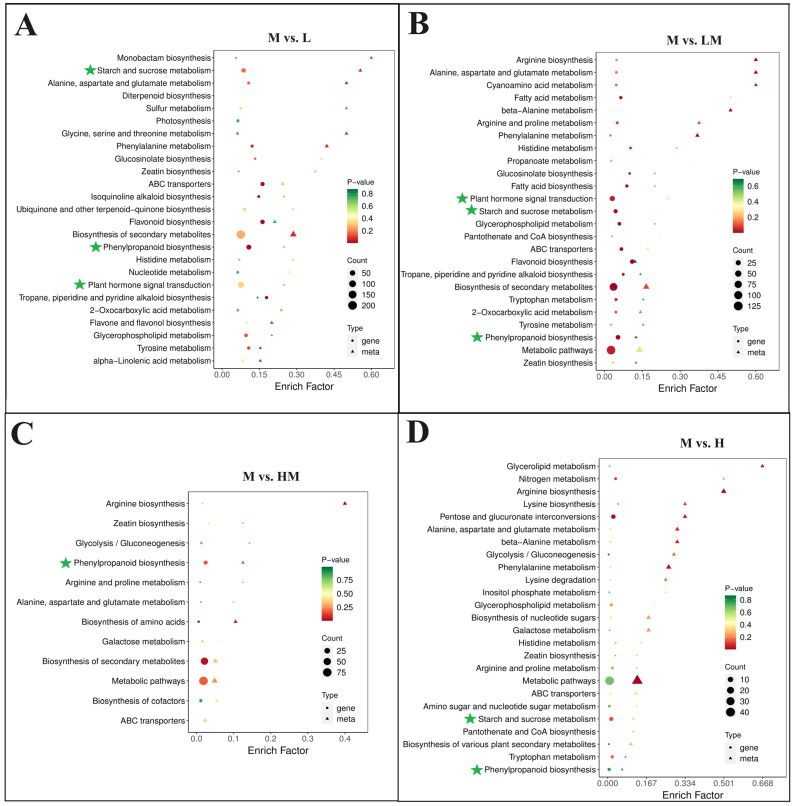
Correlation analysis between DRMs and DEGs. KEGG pathway enrichment analysis of DEGs and DRMs in (**A**) M vs. L, (**B**) M vs. LM, (**C**) M vs. HM and (**D**) M vs. H. The pathways of attention are indicated by green stars. The notations M vs. L, M vs. LM, M vs. HM and M vs. H represent 1 mM NH_4_NO_3_ versus 0.15 mM NH_4_NO_3_, 1 mM NH_4_NO_3_ versus 0.3 mM NH_4_NO_3_, 1 mM NH_4_NO_3_ versus 3 mM NH_4_NO_3_ and 1 mM NH_4_NO_3_ versus 5 mM NH_4_NO_3_, respectively.

A conjoint analysis of the transcriptome and metabolome was carried out to systematically explore the relationships between DEGs and DRMs in the cambium from the different N treatments (Figure 8A). The DEG and DRM conjoint KEGG pathway enrichment analysis showed that the DEGs and DRMs of M vs. L and M vs. LM were coenriched in the starch and sucrose metabolism, phenylpropanoid biosynthesis, plant hormone signal transduction, phenylalanine metabolism and flavonoid biosynthesis pathways. In the M vs. HM and M vs. H groups, DEGs and DRMs were also enriched in the phenylpropanoid biosynthesis pathway. The enrichment results show that the change in cambium development under the L and LM treatment (more DEG enrichment) was more obvious than that under the HM and H treatment.

**Figure 9:**
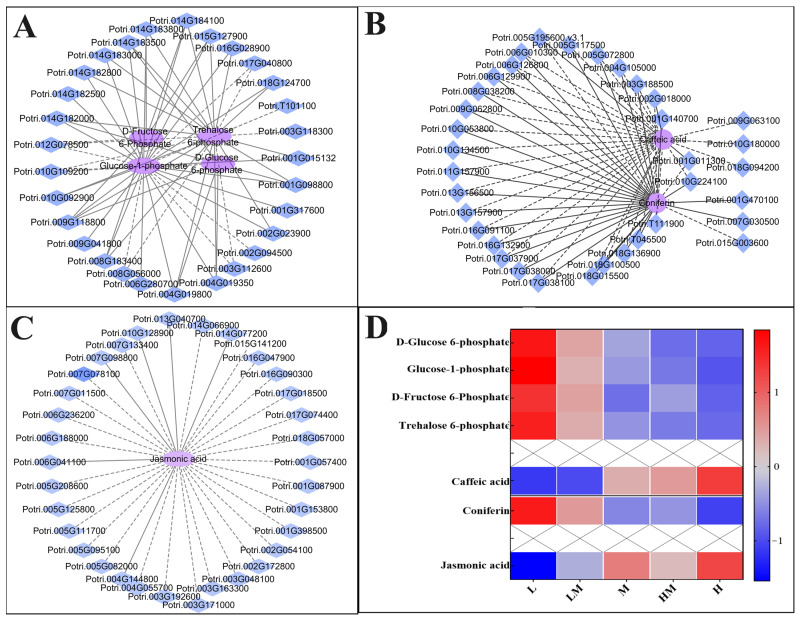
Correlation analysis between differential metabolites and differential genes. (**A**) Network of starch and sucrose metabolism pathways. (**B**) Network of phenylpropanoid biosynthesis. (**C**) Network of plant hormone signal transduction. Genes and metabolites with Pearson correlation coefficients ≥ 0.99 and *p* < 0.01 are shown. Significantly negative correlations are represented by dashed lines, and significantly positive correlations are represented by solid lines. (**D**) Content of DRMs (Z-score standardized content) in the network. Genes are represented by blue, and metabolic substances are represented by purple. The scale bar is shown on the right side. The notations L, LM, M, HM and H represent 0.15 mM NH_4_NO_3_, 0.3 mM NH_4_NO_3_, 1 mM NH_4_NO_3_, 3 mM NH_4_NO_3_ and 5 mM NH_4_NO_3_, respectively.

To reveal the correlation between the DEGs and DRMs involved in the pathways mentioned above, the networks between the DEGs and DRMs of the M vs. L group were constructed using the screening criteria of the absolute value of PCC ≥ 0.99 and *p* < 0.01. As shown in Figure 9A, four DRMs correlated with 29 DEGs in the starch and sucrose metabolism pathway, namely D-fructose 6-phosphate, glucose-1-phosphate, trehalose 6-phosphate and D-glucose 6-phosphate, whose contents were significantly accumulated in the L group (Figure 9D). Among the 29 DEGs, *glucose-1-phosphate adenylyl transferase* (*Potri.009G118800*) was significantly positively correlated with the four DRMs. The expression levels of some genes related with *cellobiases* (*Potri.004G019800* and *Potri.004G019350*), *glucan endo-1*, *3-β-d-glucosidases* (*Potri.014G183500* and *Potri.014G182000*) and *cellulase* (*Potri.001G098800*) were positively correlated with the contents of the other three DRMs except D-fructose 6-phosphate, but *Potri.010G109200* (*cellulase*) showed a negative correlation. Some genes related with *cellulases* (*Potri.008G183400* and *Potri.015G127900*) and *glucan endo-1*, *3-β-D-glucosidases* (*Potri.014G183800* and *Potri.014G184100*) were significantly positively correlated with the contents of the other three DRMs except D-glucose 6-phosphate, but *α-trehalose-phosphate synthase* (*Potri.012G078500*) was negatively correlated. The expression levels of *sucrose-phosphate synthase* (*Potri.018G124700*), *cellobiase* (*Potri.001G015132*), *4-α-glucanotransferase* (*Potri.016G028900*), *glucan endo-1*, *3-β-D-glucosidases* (*Potri.014G182800* and *Potri.014G183000*), *α-amylase* (*Potri.010G092900*) and *cellulase* (*Potri.002G023900*) were positively correlated with the contents of glucose-1-phosphate and trehalose 6-phosphate, but *glucan endo-1*, *3-β-D-glucosidase* (*Potri.008G056000*), *β-amylases* (*Potri.017G040800* and *Potri.T101100*) and *invertase* (*Potri.003G112600*) showed a negative correlation. The expression levels of *4-α-glucanotransferase* (*Potri.009G041800*), *sucrose-phosphate synthase* (*Potri.001G317600*) and *glucan endo-1*, *3-β-D-glucosidases* (*Potri.006G280700* and *Potri.014G182500*) were positively correlated with the level of glucose-1-phosphate, but *α-trehalose-phosphate synthase* (*Potri.002G094500*) was negatively correlated. The level of trehalose 6-phosphate was negatively correlated with the expression of *ADP-sugar diphosphatase* (*Potri.003G118300*).

As shown in Figure 9B,D, the contents of caffeic acid and coniferin in the phenylpropanoid biosynthesis pathway were decreased and increased under the L condition, respectively. Moreover, they were associated with 35 DEGs. *4CLs* (*Potri.003G188500*), *CCRs* (*Potri.004G105000*, *Potri.018G100500*, *Potri.001G140700*), *HCTs* (*Potri.T111900*, *Potri.006G010300*, *Potri.010G053800*), *peroxidases* (*Potri.013G156500*, *Potri.010G134500*, *Potri.T045500*, *Potri.017G037900*, *Potri.017G038000*, *Potri.017G038100*, *Potri.018G015500*, *Potri.018G136900*, *Potri.006G129900*, *Potri.005G072800*, *Potri.016G132900*), *PALs* (*Potri.010G224100*, *Potri.008G038200*, *Potri.006G126800*, *Potri.016G091100*), *CADs* (*Potri.011G157900*, *Potri.009G062800*), *C4H* (*Potri.013G157900*) and *F5H* (*Potri.005G117500*) were negatively correlated with caffeic acid content but positively correlated with coniferin. As shown in Figure 9C,D, the expression of some genes participating in the plant hormone signal transduction pathway, such as *AUX/IAAs* (*Potri.003G048100*, *Potri.006G236200*, *Potri.018G057000*), *CRE1s* (*Potri.003G171000*, *Potri.001G057400*, *Potri.005G111700*), *MYC2s* (*Potri.005G208600*, *Potri.004G055700*, *Potri.002G054100*), *TGA* (*Potri.005G082000*), *ARFs* (*Potri.002G172800*, *Potri.016G090300*), *DELLAs* (*Potri.007G133400*, *Potri.001G087900*, *Potri.017G018500*, *Potri.005G095100*, *Potri.005G125800*), *BAK1s* (*Potri.001G153800*, *Potri.003G163300*), *BRI1s* (*Potri.017G074400*, *Potri.015G141200*, *Potri.007G078100*, *Potri.001G398500*, *Potri.007G011500*), *GID1* (*Potri.003G192600*), *SAUR* (*Potri.014G066900*) and *B-ARRs* (*Potri.006G188000* and *Potri.016G047900*), were significantly negatively correlated with jasmonic acid, whose content was significantly reduced under the L treatment. The results show that the starch and sucrose metabolism, phenylpropanoid biosynthesis, plant hormone signal transduction and phenylalanine pathways and corresponding genes responded to different exogenous N concentrations.

### 2.6. Correlation between the Results of RT-qPCR and Transcriptome

**Figure 10:**
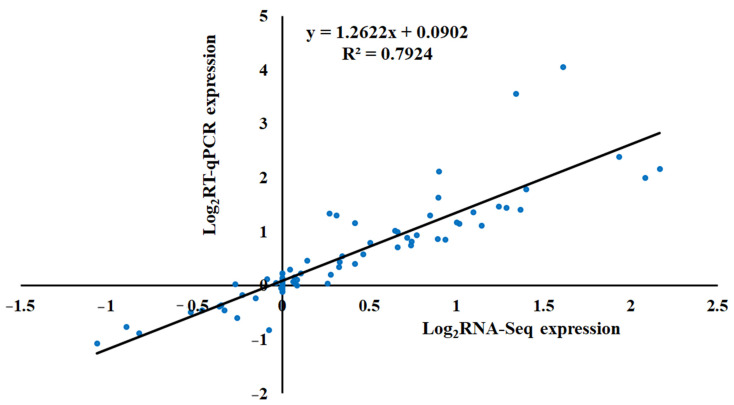
Scatter plot of correlation between gene expression levels from the transcriptome and RT-qPCR data.

To affirm the accuracy of the transcriptome data, 15 genes from pathways related to plant hormone signal transduction, phenylpropanoid biosynthesis and starch and sucrose metabolism were chosen for RT-qPCR validation (Figure 10). The results demonstrated a significant correlation (R^2^ = 0.794) between the transcriptome and RT-qPCR data, affirming the reliability of the transcriptome sequencing data.

## 3. Discussion

N is an essential macronutrient for plant growth and development [34]. N availability is a particular challenge for plant survival, so plants have evolved different metabolic regimes to deal with its fluctuation [35]. Exogenous N affects secondary structure development. For instance, the impact of elevated N concentrations on secondary growth not only results in shortened fibers but also leads to changes in the proportion of lignin monomers, resulting in a significant decrease in lignin content [24,36,37,38,39]. Stem secondary growth results from the activity of vascular cambium, which generates secondary phloem and xylem [21]. The effect of N on the development of the secondary structure of trees has been paid much attention. However, most studies focus on the regulation of N on the development of xylem, and few studies focus on the effect of N on the development of the secondary structure from the perspective of vascular cambia. In this study, different N concentrations were applied to hybrid poplar, resulting in significant differences in growth indices such as plant height and ground diameter. Specifically, significantly increased growth was exhibited for treatments with higher N concentrations, with the opposite trend under lower N concentrations. In comparison to the control, thickened fiber cell walls, decreased fiber cell areas, narrower xylem structural regions and a reduced number of layers of cambium cells were observed with anatomical methods in the L and LM treatments. Conversely, the H and HM treatments displayed contrasting effects (Figure 1). Previous studies have shown that the vascular cambium in the dormant stage exhibits thicker cell walls and fewer cell layers than those in the active stage, such as *Aesculus hippocastanum* L., *P. tomentosa* and *Cunninghamia lanceolata* [40,41,42,43]. In this study, despite the reduction in the number of cell layers, plants treated with N did not produce dormant buds, indicating that they remained in the growth stage. These findings suggest that low N levels impact the secondary growth of poplar, and the self-protection mechanism of the plant is triggered, leading to entry into the dormant stage only under prolonged exposure to low or extremely low N conditions.

To elucidate the impact of N on the secondary development of hybrid poplar, our study analyzed cambial changes under different N treatments at the transcriptional and metabolic levels. The development of vascular cambium is a highly complex process regulated by many factors [9,10,11,12]. As one of the factors regulating the development of vascular cambium, hormones have been paid much attention. Aux plays a central role in regulating the initiation and maintenance of the procambium [9,44]. *MP/ARF5* functions as an Aux activator, positively regulating the quantity of vascular initial cells by inducing the expression of *PIN1*, an Aux transport gene [9,44,45,46,47]. The hybrid aspen *PIN-FORMED* genes *PttPIN1* and *PttPIN12*, which are expressed in the cambial meristem and extended xylem derivatives, play key roles in polar auxin transport in stems [48]. The influx carrier *AUX1/LAX* affects the development of the vascular cambium, with its mutation resulting in a reduction in vascular bundle production and increased spacing between bundles. Mutating the amino acids in the poplar Aux inhibitor IAA3 made it unresponsive to Aux, thereby inhibiting the periclinal division of the vascular cambium [49]. *WOX4*, as a downstream transcription factor of auxin, has the capability to activate the division activity of the formative layer. Auxin-activated *MP/ARF5* directly inhibits the expression of *WOX4* and autonomously restricts the quantity of vascular cambium stem cells [50]. In the suppressed expression lines of *PttWOX4a/b* in *Populus* trees, the division activity of the vascular cambium is diminished, leading to a reduction in secondary xylem formation [51]. The poplar *ARF7* serves as a molecular bridge, integrating the Aux and GA signaling pathways and regulating cambium activity [15]. In *P. tomentosa*, *PtoHB7* and *PtoHB8*, downstream participants of *Aux/IAA9*-*ARF5*, are involved in the Aux-mediated differentiation regulation of xylem cells [52]. These studies confirm the significance of Aux in the formation and activity of the vascular cambium. CK also plays a central role in regulating the activity of vascular cambium division. The overexpression of the *Arabidopsis* cytokinin *CKX2* gene, involved in catabolism, in poplar disrupts CK signaling, leading to a reduction in the number of vascular cambium cell layers and the inhibition of secondary growth in poplar [14]. The overexpression of *PtoCYCD3:3* (cell division cycle regulator cyclin) enhances the activity of the vascular cambium of poplar, thereby enhancing secondary growth [53]. Therefore, CK is a key plant hormone regulating the radial direction of stem thickening and vascular cambium activity. Aux and CK can regulate each other; for example, Aux flows to cells surrounding the cambium to activate the suppressor of CK signaling (*AHP6*) and reduces CK signaling in these cells [54,55]. The signal level of GA was highest in the differentiated xylem region and decreased toward the vascular cambium. The enhancement of vascular cambium division activity was observed when GA was applied locally to the stems of some angiosperms (*Fraxinus mandshurica* var. *japonica*, Quercus *mongolica* var. *grosseserrata*, *Kalopanax pictus* and *P. sieboldii*) [56]. Overexpressing GA 20-oxidase, a key enzyme in the GA biosynthetic pathway, in hybrid aspen significantly enhances the activity of the vascular cambium and increases the quantity of xylem fibers [57]. In *Populus*, bioactive GA peaks are detected in developing xylem [58]. The constitutive or xylem-specific expression of *PdGA20ox1* from the pine *Pinus densiflora* encoding GA20 oxidase, a key enzyme that catalyzes the production of bioactive GA forms from GA12, increases xylem width and cell number in transgenic hybrid poplar [59]. Increased GA levels improve cambial growth in transgenic poplar [60], suggesting that GA also plays a vital role in regulating vascular cambial activity. Similarly, overexpressing GA20ox and the GA receptor gene *GID1* in poplar results in enhanced cambial proliferation and secondary growth [61]. In poplar, GA and Aux interact to regulate the development of vascular cambium, and DELLA, RGL1, ARF7 and IAA9 form a ternary complex to mediate the concatenation between Aux and GA in poplar stems [15]. BR can promote the division of procambia. In *Arabidopsis*, the gain-of-BR signal mutants *bin2* and *bzr1-1D* show significant increases in the number of vascular bundles, whereas the loss-of-function mutants *bri1-116* and *cpd* show significant decreases [9]. In addition to inducing cambium initiation in the primary growth stage, BR signaling also plays a role in secondary growth. Exogenously applied BR can reduce lignification and alter cell wall carbohydrate biosynthesis in the secondary xylem of *Liriodendron tulipifera* trees [62]. In poplar, the gene encoding the *BEE3* basic helix–loop–helix transcription factor is induced by BR, and when overexpressed, it results in enhanced secondary xylem formation [63]. The overexpression of the BR biosynthesis gene *BR6OX* in poplar shows increased secondary xylem formation [64]. When BR biosynthesis is inhibited in *Populus*, secondary xylem differentiation decreases, suggesting that BR is a key regulator of secondary growth by acting to stimulate cell differentiation [65]. In addition, there is a regulatory correlation between the BR signal and Aux signal, and the BR signal and Aux coordinate to regulate the formation of vascular bundles [9]. It was demonstrated that the concentrations of GA, jasmonic acid and abscisic acid in wood are significantly lower under low N conditions in *P.* × *canescens*, in contrast to the significantly increased concentrations of salicylic acid and Aux [66]. In this study, exogenous N treatment not only caused changes in the development of vascular cambium (Figure 1) but also affected the expression levels of some genes evolved in the plant hormone signaling pathway. For example, *AUX/IAAs*, *ARFs*, *A-ARRs*, *B-ARRs*, *CRF1s*, *AHPs*, *GID1s*, *DELLAs*, *TFs*, *BAK1s*, *BRI1s*, *JAZs* and *MYC2s* were significantly highly expressed under low N. In addition, the JA content was significantly reduced under L conditions (Figure 3 and Figure 9). Interestingly, these changes are significant only within the comparative groups of M vs. L and M vs. LM. This phenomenon can be attributed to the inference that the concentrations of H and HM were inadequate to attain elevated N levels conducive to plant growth. Conversely, L and LM were able to sustain low N conditions. These findings suggest that the process of exogenous N application on wood development may be due to the fact that N’s effect on the hormone signaling process affects cambium development and further changes wood development.

The ubiquitin–proteasome system (UPS) selectively degrades proteins involved in the developmental regulation of the plant vascular cambium [11]. The degradation process through UPS involves two steps: Proteins are initially marked by a covalent link of ubiquitin, followed by degradation by the 26S proteasome complex. The transfer of ubiquitin to target proteins requires ubiquitin-protein ligase enzymes (E3). E3 enzymes play a pivotal role in substrate specification during the ubiquitination reaction. The Anaphase-Promoting Complex/Cyclosome (APC/C) is known to be the largest E3 identified to date, whose composition involves the participation of RING-finger proteins and cell cycle proteins [67]. In the expression profile analysis of DEGs (Figure 5B), several ubiquitin-related genes were identified, such as *Potri.011G067800* (*APC/C13*), *Potri.017G122800* (E3 ubiquitin-protein ligase gene), *Potri.019G083800* (ubiquitin-conjugating-like enzyme gene), *Potri.009G005700* (zinc finger family gene) and *Potri.009G049600* (ubiquitin-conjugating enzyme gene), exhibiting significantly lower expression in L and LM conditions. Conversely, *Potri.011G052600* (ubiquitin-conjugating enzyme family gene) and *Potri.004G043500* (ubiquitin-conjugating enzyme family gene) showed significantly higher expression in L and LM conditions. In *Arabidopsis*, the loss of *APC/C* genes affects the normal distribution of the hypocotyl vascular tissue and causes excessive proliferation of the cambium, leading to the formation of excess lignified sclerenchyma [68]. In poplar, it was demonstrated that ubiquitin receptor proteins regulate the stability of the key transcription factor *WOX4* through ubiquitination-dependent modifications in the cambium, thereby participating in the regulation of vascular cambium activity and wood formation [11]. In this study, under L and LM conditions, there was a significant increase in the thickness of the fiber cell walls and lignin content, resembling the phenotype of *APC/C* mutants. Additionally, alterations in the expression patterns of certain ubiquitin-related genes were observed under low N conditions. Thus, it is speculated that N-induced changes in the cambial region may be related to the selective degradation of ubiquitin, and some genes of the ubiquitin degradation pathway may play roles in this process. The activity of cambium and the SAM is essential for maintaining the uncertain growth of cells. Therefore, some key genes or homologous genes of the SAM are also expressed in cambium [19]. In the SAM, transcription factors such as *WUS*, which specifies the fate of meristematic cells, and *PtaRHE1*, which is involved in the transition from primary to secondary growth in hybrid poplar, have been identified [6,20,21]. In the expression profile analysis of DEGs (Figure 5), the expression patterns of their corresponding homologous genes, *Potri.006G094600* and *Potri.006G245400*, varied with different N concentrations. Additionally, the expression patterns of genes related to starch and sucrose metabolism (*Potri.003G112600*, *Potri.014G118400*, *Potri.009G062800*, *Potri.018G100500*, *Potri.009G063100*, *Potri.019G038200*, *Potri.016G031100*, *Potri.005G093200* and *Potri.005G117500*) were also changed under different N concentrations. The homologous genes of these genes have been shown to play a role in the transition from primary to secondary growth in hybrid aspen trees [20,21], suggesting that N can influence the process of transitioning from primary to secondary growth.

In the combined analysis of transcriptomics and metabolomics (Figure 8), genes and metabolites associated with the starch and sucrose metabolism, phenylpropanoid biosynthesis and plant hormone signal transduction pathways were identified in the cambium of M vs. L and M vs. LM. The expression levels and metabolite contents showed significant correlations. The networks of the phenylpropanoid biosynthesis and starch and sucrose metabolism pathways (Figure 9A,B) illustrate that the influence of N on wood development emerges during cambial development. This impact extends beyond affecting lignin content to influencing non-structural carbon content as well. Additionally, the enrichment of DEGs and DRMs within the plant hormone signal transduction pathway, along with alterations in the expression levels of ubiquitin-related genes detected in the biosynthesis of secondary metabolites (Figure 5B and Figure 9C), suggests that nitrogen is not only a factor that influences cambial development but may also achieve this effect by regulating the plant hormone signal transduction pathway and the ubiquitin–proteasome degradation pathway. In conclusion, the application of exogenous N induces variations in cambial development, representing one of the factors of wood development. These differences are associated with several genes within the starch and sucrose metabolism, phenylpropanoid biosynthesis and plant hormone signal transduction pathways and ubiquitin-related genes. Although these genes may play crucial roles in the regulation of cambial development under exogenous N, their underlying mechanisms require further investigation.

## 4. Materials and Methods

### 4.1. Plant Materials and Treatments

The experimental material, *P.* × *xiaohei* T. S. Hwang et Liang, was sourced from the State Key Laboratory of Tree Genetics and Breeding at Northeast Forestry University in Harbin, China. Seedlings were initially rooted in water and subsequently transplanted into vermiculite, where they were cultured for 42 days in a greenhouse under controlled conditions: a 16 h light/8 h dark photoperiod at a temperature of 22 ± 2 °C. And the seedlings received irrigation every two days, utilizing a Long Ashton (LA) nutrient solution with a N concentration fixed at 1 mM NH_4_NO_3_ [69]. At the initiation of N treatments, the apical region of each plant was labeled to differentiate the shoots developed during this phase. Subsequently, the seedlings underwent a 28-day treatment with an LA nutrient solution of varying N concentrations (0.15 mM NH_4_NO_3_ (L), 0.3 mM NH_4_NO_3_ (LM), 1 mM NH_4_NO_3_ (M), 3 mM NH_4_NO_3_ (HM) and 5 mM NH_4_NO_3_ (H)) [70]. The 1 mM NH_4_NO_3_ group was considered the control [69]. Three biological repeats were conducted for each treatment. The bark of each plant was separated with scissors and tweezers, and the cambium was scraped (once) with a scalpel after freezing in liquid nitrogen. The whole process of separating the cambium was carried out in liquid nitrogen. RNA was extracted immediately after sampling for machine analysis of the transcriptome and metabolome. In addition, plants from each treatment were dried for the determination of cellulose, lignin and hemicellulose contents.

### 4.2. Determination of Growth Indices and NH_4_^+^ and NO_3_^−^ Contents

Plant height and ground diameter were measured with a ruler and vernier caliper, respectively. The NH_4_^+^ and NO_3_^−^ contents were determined using methods described previously [71].

### 4.3. Wood Composition and Microscopy Assay

The lignin content and cellulose were determined according to a method described previously [72,73]. Hemicellulose was determined with a hemicellulose content reagent kit (Suzhou Kming Biotechnology Co., Ltd., Suzhou, China). The secondary structure of internode stems (5 cm stem in length above the mark for N treatments) under a microscope were observed with toluidine blue and phloroglucinol–hydrochloric acid staining [74]. For scanning electron microscopy (SEM), sections of fresh stem internodes were coated with gold (Au), transferred to an SEM (S-4800; Hitachi, Tokyo, Japan) chamber and imaged to analyze the fiber cell wall thickness and area.

### 4.4. Transcriptome Sequencing and Analysis

Total RNA was extracted from the cambium using CTAB [75]. RNA degradation and contamination were monitored on 1% agarose gels. The purity, concentration and integrity of RNA were assessed using the NanoPhotometer spectrophotometer (IMPLEN, CA, USA), the QubitR RNA Assay Kit in the QubitR 2.0 Fluorometer (Life Technologies, CA, USA) and the RNA Nano 6000 Assay Kit of the Bioanalyzer 2100 system (Agilent Technologies, CA, USA), respectively. We ensured that the RNA Integrity Number (RIN) for each sample was greater than 7 before proceeding with library construction [76]. The NEBNext Ultra RNA Library Prep Kit for Illumina (California, USA) was employed for library construction, and sequencing took place on an Illumina HiSeq platform at Metware BioTech Co. (Wuhan, China). Subsequently, clean reads were extracted from the original data and aligned to the reference genome sequence of *Populus* (http://ftp.ensemblgenomes.org/pub/plants/release-52/fasta/populus_trichocarpa/dna/, accessed on 3 January 2024) using HISAT2 software (version: 2.2.1) [77]. Only reads with a perfect match were further analyzed and annotated based on the reference genome. Gene function was annotated based on the following databases using the HMMER webserver (https://www.ebi.ac.uk/Tools/hmmer, accessed on 2 September 2023): Kyoto Encyclopedia of Genes and Genomes (KEGG), National Center for Biotechnology Information non-redundant protein sequences (Nr), a manually annotated and reviewed protein sequence database (SwissProt), an automated annotation and review database of protein sequences (TrEMBL), Eukaryotic Orthology Groups (KOG), Gene Ontology (GO) and Protein family (Pfam). The gene expression levels were calculated using the fragments per kilobase of transcript per million mapped reads (FPKM) mapped method. EBSeq2 (version: 1.22.1) was used to screen differentially expressed genes (DEGs). Following differential analysis, the false discovery rate (FDR) was obtained by applying the Benjamini–Hochberg method for multiple hypotheses testing correction to the hypothesis test probabilities (*p* values). The thresholds of the DEGs were |log_2_ FC (fold change)| ≥ 1 with FDR < 0.05. The enrichment analysis was performed based on the hypergeometric test. For KEGG, the hypergeometric distribution test was performed with the unit of the pathway; for GO, it was performed based on GO terms. Correlation analysis and principal component analysis (PCA) were conducted using the built-in functions cor and prcomp (with scale = True), respectively, in R software (version: 4.3.2, www.r-project.org/, accessed on 3 January 2024). The Z-score normalization of the FPKM of DEGs was performed using the scale function in R software for subsequent analysis. The ComplexHeatmap package and ggplot2 package in R software were used to generate the heatmap and volcano plot, respectively.

### 4.5. Metabolomics Analysis

The cambiums from each of the five treatment groups underwent dehydration using a vacuum freeze-dryer and were subsequently pulverized into a powder. After weighing 50 mg of freeze-dried powder, three extractions were carried out using 1.2 mL of 70% methanol at 4 °C for 60 min each. The samples underwent centrifugation at 12,000 rpm for 10 min. The resulting supernatant was filtered through a 0.22 µm pore size membrane (ANPEL, Shanghai, China). An analysis of the filtrate was conducted using a UPLC-ESI-MS/MS system (UPLC: Nexera X2 system, Shimadzu, Kyoto, Japan; MS, 4500 Q TRAP, Applied Biosystems, Waltham, MA, USA) at Metware BioTech Co. (Wuhan, China). The UPLC-MS/MS system acquired linear ion trap and triple quadrupole scans. And they were connected to an ESI Turbo Ion-Spray interface, controlled by Analyst 1.6.3 software (AB Sciex, Framingham, MA, USA) and operated in both positive and negative ionization modes. Metabolite identification was based on the parametric values (*m*/*z* data, retention time and fragmentation partners) and was compared with the self-built database (Metware) for the annotation results. PCA and a partial least squares discriminant analysis (PLS-DA) were conducted to identify the differential metabolites (DRMs), with a fold change ≥ 2 or a fold change ≤ 0.5, and the metabolites had a VIP ≥ 1. PCA and PLS-DA were performed using the prcomp function and the MetaboAnalystR package in R software, respectively.

### 4.6. Conjoint Analysis of the Transcriptome and Metabolome

DEGs and DRMs were identified via a transcriptome and metabolome analysis. According to the annotation information of metabolites in the KEGG database, the corresponding metabolite transcripts were screened. The metabolites and associated transcripts were then mapped to the relevant metabolic KEGG pathways. The cor function in R software was used to calculate the Pearson correlation coefficients (PCCs) between DEGs and DRMs. The network diagrams were plotted using DEGs and DRMs (PCCs ≥ 0.99 and *p* < 0.01) with the Cytoscape tool (version: 3.8).

### 4.7. Real-Time Quantitative PCR (RT-qPCR) Analysis

Total RNA extracted from cambium was reverse transcribed into cDNA with the PrimeScript TM RT reagent Kit (containing gDNA Eraser) (Takara, Dalian, China). RT-qPCR was performed with LightCycler 480 II (Roche, Basel, Switzerland), and the 2^−ΔΔCT^ method was used to calculate the relative expression level [78]. The primer sequences are listed in Table S3.

### 4.8. Statistical Analysis

SPSS 22.0 software (SPSS Inc., Chicago, IL, USA) was used to conduct a one-way ANOVA and Duncan’s test for the data.

## 5. Conclusions

In this study, the impacts of different N concentrations on the cambium development of *Populus* were compared. The results indicate that the L and LM treatments led to a decrease in cellulose content and an increase in lignin and hemicellulose content in comparison to the M treatment. Additionally, the secondary xylem became thinner, the number of cambial layers decreased, and the cell wall thickness increased under the L and LM treatments, whereas the HM and H treatments exhibited the opposite effects. The transcriptome analysis showed that plant hormone signal transduction, phenylpropanoid biosynthesis and starch and sucrose metabolism were enriched. Furthermore, the expression patterns of DEGs suggested that, in addition to genes related to plant hormone signal transduction, starch and sucrose metabolism and phenylpropanoid biosynthesis, certain ubiquitin-related genes may also play a role in the process of nitrogen affecting wood development. The metabolome analysis revealed significant differences in the abundance of substances belonging to flavonoids, lignans and coumarins, and saccharides. The combined analysis of transcriptomics and metabolomics indicated that, under the L treatment, D-fructose 6-phosphate, glucose-1-phosphate, trehalose 6-phosphate, D-glucose 6-phosphate, caffeic acid and coniferin, which are associated with non-structural carbon and structural carbon, respectively, are significantly correlated with 29 and 35 DEGs. These DEGs may be involved in N-regulated processes of cambial development into phloem and xylem, but their biological functions require further investigation. This study provides new insights into the impact of exogenous N on wood development.

## Figures and Tables

**Table 1:** Statistics of the annotated transcripts in the seven databases.

Database	KEGG	Nr	Swissport	TrEMBL	KOG	GO	Pfam	Total
Number	21,624	36,551	27,253	37,756	19,089	31,083	29,829	38,285

KEGG: Kyoto Encyclopedia of Genes and Genomes; Nr: National Center for Biotechnology Information non-redundant protein sequences; SwissProt: A manually annotated and reviewed protein sequence database; TrEMBL: An automated annotation and review database of protein sequences; KOG: Eukaryotic Orthology Groups; GO: Gene Ontology; Pfam: Protein family.

**Table 2:** Statistics of differentially regulated metabolites.

Group	M vs. L	M vs. LM	M vs. HM	M vs. H
Up	195	120	40	67
Down	164	70	41	71

The notations M vs. L, M vs. LM, M vs. HM and M vs. H represent 1 mM NH_4_NO_3_ versus 0.15 mM NH_4_NO_3_, 1 mM NH_4_NO_3_ versus 0.3 mM NH_4_NO_3_, 1 mM NH_4_NO_3_ versus 3 mM NH_4_NO_3_ and 1 mM NH_4_NO_3_ versus 5 mM NH_4_NO_3_, respectively.

## Data Availability

All experiments and data are available in this article and the Supplementary Materials.

## Supplementary Materials

The following supporting information can be downloaded at https://www.mdpi.com/article/10.3390/ijms25021017/s1.
