# Supplementary material for: Network Analysis of Metabolome and Transcriptome Revealed Regulation of Different Nitrogen Concentrations on Hybrid Poplar Cambium Development
Source: Int J Mol Sci. 2024 Jan 13;25(2):1017. doi: 10.3390/ijms25021017 (PMC10816006; doi:10.3390/ijms25021017)
Supplement: Supplementary file 1 [file ijms-25-01017-s001.zip › Fig S2.pdf]

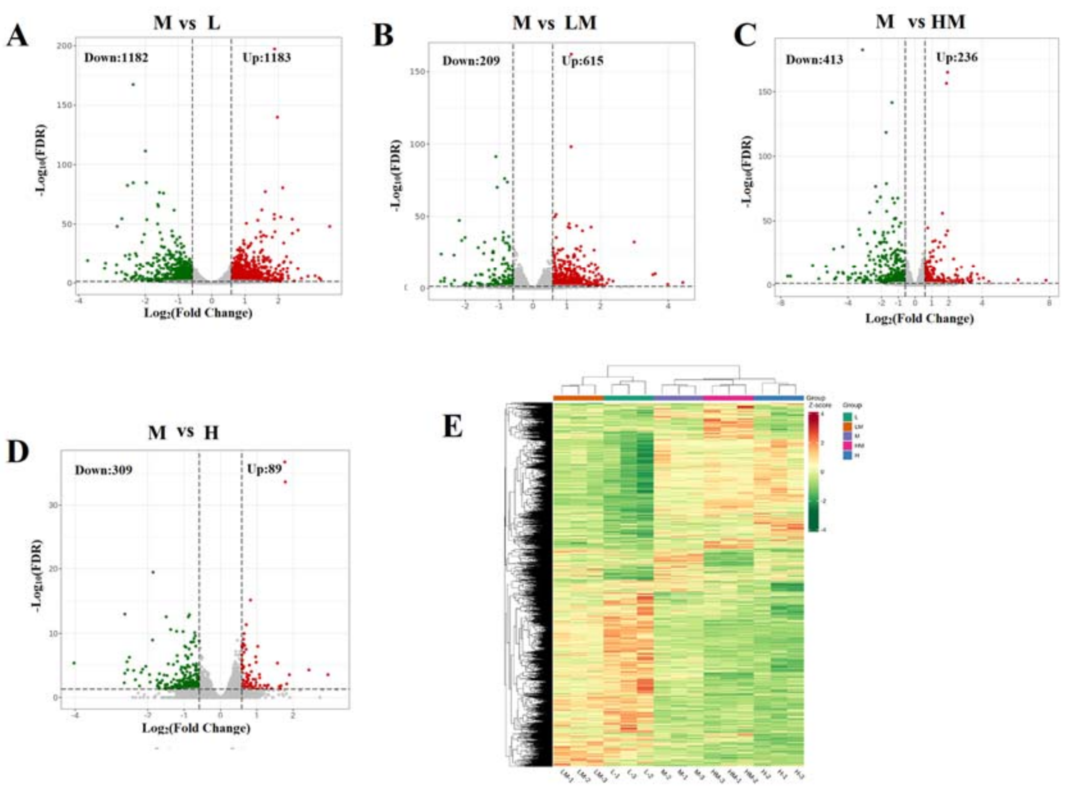

Fig. S2. Statistical and expression profile analysis of DEGs. Statistics of DEGs in A. M vs L; B. M vs LM; C. M vs HM; D. M vs H; E. Expression profile analysis of all DEGs.
