# Supplementary material for: Network Analysis of Metabolome and Transcriptome Revealed Regulation of Different Nitrogen Concentrations on Hybrid Poplar Cambium Development
Source: Int J Mol Sci. 2024 Jan 13;25(2):1017. doi: 10.3390/ijms25021017 (PMC10816006; doi:10.3390/ijms25021017)
Supplement: Supplementary file 1 [file ijms-25-01017-s001.zip › Fig S3.pdf]

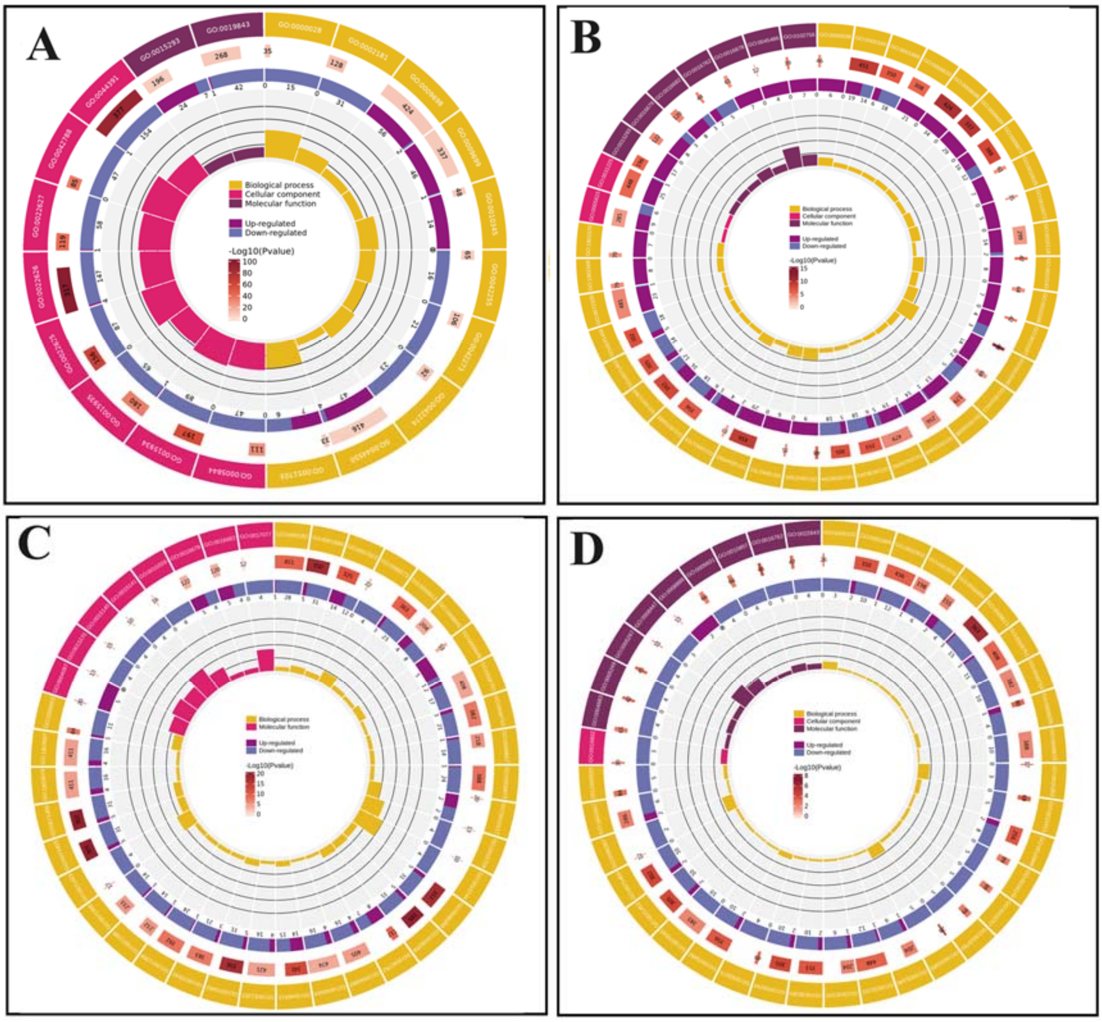

Fig.S3 GO enrichment analysis of DEGs. A. M vs L; B. M vs LM; C. M vs HM; D. M vs H DEGs GO enrichment cycle diagram. The outermost circle is ID, and the Ontology to which the ID belongs is distinguished by color; The second circle is the total number of genes in the term, represented by color -log10 (*P*-value); The third circle is the number of up/down-regulated genes; The fourth circle is Rich factor, and the innermost circle is legend information.
