# Supplementary material for: Network Analysis of Metabolome and Transcriptome Revealed Regulation of Different Nitrogen Concentrations on Hybrid Poplar Cambium Development
Source: Int J Mol Sci. 2024 Jan 13;25(2):1017. doi: 10.3390/ijms25021017 (PMC10816006; doi:10.3390/ijms25021017)
Supplement: Supplementary file 1 [file ijms-25-01017-s001.zip › Fig S4.pdf]

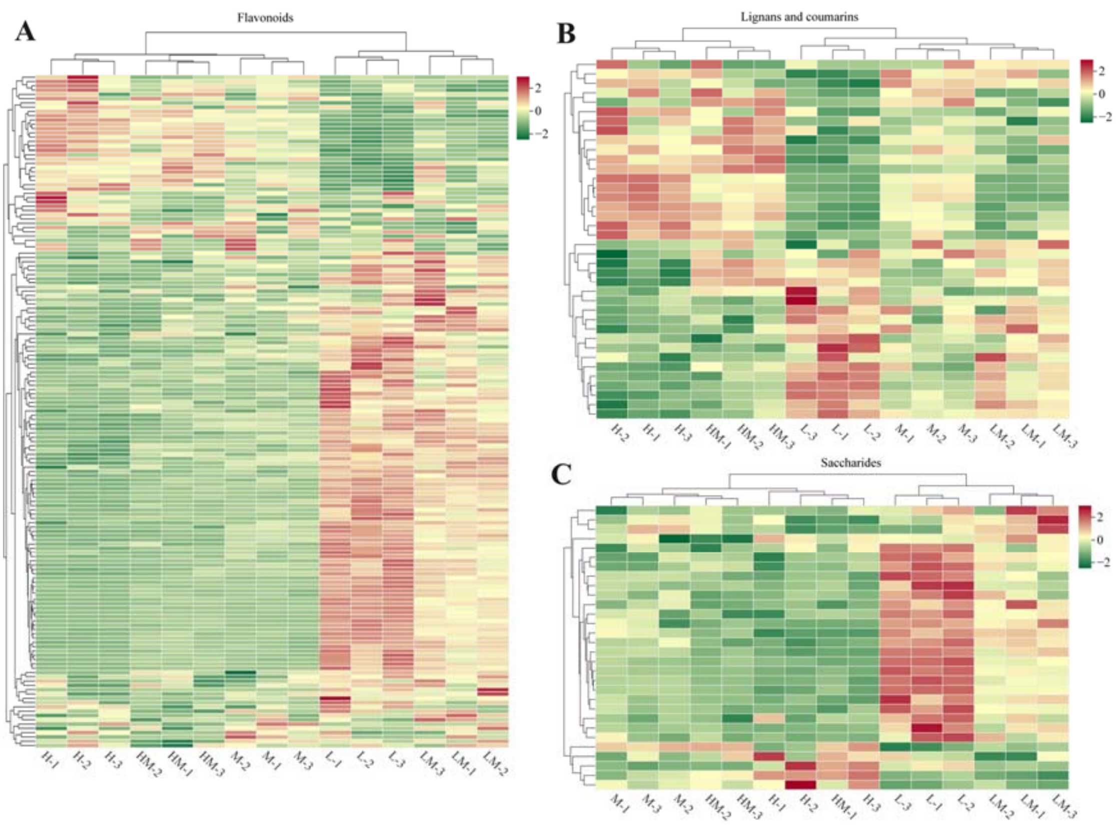

Fig. S4 Content changes of the differentially regulated flavonoids (A), lignans and coumarins (B) and (C) saccharides.
