# Supplementary material for: Network Analysis of Metabolome and Transcriptome Revealed Regulation of Different Nitrogen Concentrations on Hybrid Poplar Cambium Development
Source: Int J Mol Sci. 2024 Jan 13;25(2):1017. doi: 10.3390/ijms25021017 (PMC10816006; doi:10.3390/ijms25021017)
Supplement: Supplementary file 1 [file ijms-25-01017-s001.zip › Table S1.pdf]

Table S1 The data of height, diameter, numbers of internode/leaves, NH4+, NO3-, the contents of lignin, cellulose, and hemicellulose.

Sample	Height (cm)	Diameter (mm)	Interganglion/ Leaves number	Ammonium nitrogen content (μg/g)	nitrate nitrogen concentration (μg/g)	Lignin content (%)	Cellulose content (%)	Hemicellulose content (%)
L	80.5	4.08	26/25	3.692588	598.9481	26.84704	39.27273	17.16421
	80	4.43	27/26	4.056389	612.0973	27.0102	38.24242	17.12211
	81	4.46	26/25	3.692588	589.0861	27.98912	38.48485	17.03789
	80	4.48	26/25			26.76547	39.15152	
	82	4.57	24/23				39.75758	
LM	83	4.52	28/27	7.421555	1144.642	25.46023	40.18182	16.84842
	82.5	4.53	26/25	6.693952	1144.642	26.03127	40.36364	16.93263
	84	4.49	28/27	6.693952	1147.929	25.29708	40.84848	16.91158
	85	4.48	28/27			25.13392	39.81818	
	84.4	4.59	27/26				40.12121	

	**86.3**	**4.66**	**29/28**	**12.51478**	**1664.037**	**20.81033**	**42.36364**	**15.98526**
	**86.5**	**4.68**	**29/28**	**13.33333**	**1815.253**	**21.78926**	**42.66667**	**16.23789**
**M**	**87**	**4.76**	**29/28**	**13.33333**	**1805.391**	**21.05506**	**42**	**16.42737**
	**86.5**	**4.69**	**30/29**			**21.70768**	**42.42424**	
	**88**	**4.63**	**31/30**				**44**	
	**90**	**4.81**	**33/32**	**16.42565**	**1969.757**	**18.85248**	**43.51515**	**15.98526**
	**91**	**4.91**	**32/31**	**16.1528**	**2032.216**	**19.17879**	**43.21212**	**15.75368**
**HM**	**91.5**	**4.9**	**32/31**	**15.33424**	**1927.022**	**19.26037**	**43.69697**	**15.92211**
	**92**	**4.96**	**32/31**			**18.68933**	**43.09091**	
	**91**	**4.89**	**33/32**				**43.87879**	
	**97**	**5.02**	**33/32**	**17.7899**	**1996.055**	**19.91298**	**46**	**15.73263**
	**96**	**4.99**	**34/33**	**17.7899**	**2028.928**	**20.23929**	**45.63636**	**15.92211**
**H**	**95**	**5.05**	**34/33**	**19.42701**	**2081.525**	**19.91298**	**45.69697**	**15.62737**
	**93**	**5.03**	**33/32**			**20.15772**	**45.09091**	
	**92**	**5.18**	**34/33**				**44.9697**	
