# Supplementary material for: Network Analysis of Metabolome and Transcriptome Revealed Regulation of Different Nitrogen Concentrations on Hybrid Poplar Cambium Development
Source: Int J Mol Sci. 2024 Jan 13;25(2):1017. doi: 10.3390/ijms25021017 (PMC10816006; doi:10.3390/ijms25021017)
Supplement: Supplementary file 1 [file ijms-25-01017-s001.zip › Table S2.pdf]

**Table S2 Statistics of the data generated by the transcriptome sequencing**

| Sample | Reads | Q30 (%) | Clean Reads | Clean Reads (%) | Mapped Reads | Mapped Reads (%) |
|---|---|---|---|---|---|---|
| L1 | 72450762 | 93.45 | 66073712 | 91.20 | 57422777 | 86.91 |
| L2 | 56381628 | 93.41 | 53342500 | 94.60 | 46895904 | 87.91 |
| L3 | 63665920 | 93.60 | 59348140 | 93.22 | 51850102 | 87.37 |
| LM1 | 71196840 | 93.60 | 66226606 | 93.02 | 57819332 | 87.31 |
| LM2 | 59676280 | 93.20 | 55606620 | 93.19 | 48404720 | 87.05 |
| LM3 | 54808230 | 93.04 | 51414164 | 93.81 | 44890399 | 87.31 |
| M1 | 61149972 | 93.42 | 56830012 | 92.94 | 49361326 | 86.86 |
| M2 | 53828280 | 93.09 | 49746932 | 92.42 | 42944848 | 86.33 |
| M3 | 70223978 | 93.58 | 64041340 | 91.20 | 55648237 | 86.89 |
| HM1 | 60016574 | 93.89 | 55375358 | 92.27 | 48525787 | 87.63 |
| HM2 | 59726742 | 94.05 | 55123066 | 92.29 | 48369413 | 87.75 |
| HM3 | 53454620 | 93.29 | 49866530 | 93.29 | 43519347 | 87.27 |
| H1 | 61523686 | 93.28 | 56956508 | 92.58 | 49679777 | 87.22% |
| H2 | 66583494 | 93.43 | 63092836 | 94.76 | 55189741 | 87.47% |
| H3 | 59603740 | 93.30 | 55324322 | 92.82 | 48429139 | 87.54% |
