# Supplementary material for: Network Analysis of Metabolome and Transcriptome Revealed Regulation of Different Nitrogen Concentrations on Hybrid Poplar Cambium Development
Source: Int J Mol Sci. 2024 Jan 13;25(2):1017. doi: 10.3390/ijms25021017 (PMC10816006; doi:10.3390/ijms25021017)
Supplement: Supplementary file 1 [file ijms-25-01017-s001.zip › Table S3.pdf]

Table S3 Primer sequences for qRT-PCR analysis.

Locus name (POPTR)	Primers (5′-3′)	Product size (bp)	Annealing temp (°C)
	F: GGCTGAAGGAGAGATGGTGAGG		60
Potri.002G044900	R: ACTGGAGGCCATCCCACAAC	192	
	F: GAGAGAGGGGTTTCTTCCAAACTG		60
Potri.001G057400	R: CCCTTATTCCCGCCCATTGCT	164	
	F: CACTGGAGCCCAAGCAACTACA		60
Potri.006G188000	R: AATATCGGAGGGCTGCTTGTGG	169	
	F: CTTGCCGGAGGCATTCCAGT		60
Potri.003G192600	R: GTGGTGCTGCATGTCCCACT	184	
	F: CCCTTCATCCAAACGGGTCAGT		60
Potri.006G021400	R: GCAAGAGCCCAAGCAACCTG	128	
	F: ACGCCCTGATGAATGGTGAGA		60
Potri.008G038200	R: ACCTGCATTCCGTGATCCTGT	171	
	F: AGGGTTGAAGTGCGCCATAGA		60
Potri.013G157900	R: TGCAGCGACGTTGATGTTCTC	103	
	F: GGTGTCGGCCATGCATCCTA		60
Potri.009G063100	R: CCTATGCAACTCCCACCCACT	150	
	F: GCGCATTCAGTGATCGGTTCC		60
Potri.018G015500	R: TGTGGGCCACTAGGTTTCGGT	195	
	F: ACGCCCTTGATTTGGCTGTTG		60
Potri.005G117500	R: TAAAATGGACAGACCACGCGCT	166	
	F: ATGGCTGAGGGTGTCAAGGTG		60
Potri.001G015132	R: ACCGAGCTGAGAGTTTTGGGA	148	
	F: GCCACAATCCTCCCTGATGG		60
Potri.003G112600	R: GGATCATCGTGGTCTGCTGG	104	
	F: TAGCAGCAACCACCAACACCAT		60
Potri.014G183800	R: CTTTGTTGCTACCACTGCTGATGT	117	
	F: ATTCGCAAGAGGGCAGGTGG		60
Potri.015G127900	R: GAGCCGCGGTGGTGTATCTT	108	
	F: TCCGGCACAAGATTCGCTCA		60
Potri.002G023900	R: AGTAAGGGGCCTCTTTTCACTCA	151	
Potri.017G144700.1（PtrUGP2）	F: GCTTGATAGTCTAAAGGTGGTCGGT	156	60
	R: TAGAGGTCCTCCGGGCCATT		
Potri.0005G22060.1（PtrUBQ7）	F: GGAACGGGTTGAGGAGAAAGAAG	135	60
	R: GCAAGAACAAGATGAAGCACAGAGC		
